# Acupuncture Needling, Electroacupuncture, and Fire Needling Improve Imiquimod-Induced Psoriasis-Like Skin Lesions through Reducing Local Inflammatory Responses

**DOI:** 10.1155/2019/4706865

**Published:** 2019-07-29

**Authors:** Yan Wang, Yuanbo Fu, Lu Zhang, Jing Fu, Bin Li, Luopeng Zhao, Tingting Di, Yujiao Meng, Ningfei Li, Jianning Guo, Ping Li, Jingxia Zhao

**Affiliations:** ^1^Beijing Hospital of Traditional Chinese Medicine, Capital Medical University, Beijing Institute of Traditional Chinese Medicine, Beijing Key Laboratory of Clinic and Basic Research with Traditional Chinese Medicine on Psoriasis, No. 23, Art Gallery Back Street, Dongcheng District, Beijing 100010, China; ^2^Acupuncture and Moxibustion Department, Beijing Hospital of Traditional Chinese Medicine, Capital Medical University, Beijing Key Laboratory of Acupuncture Neuromodulation, No. 23, Art Gallery Back Street, Dongcheng District, Beijing 100010, China; ^3^Beijing University of Chinese Medicine, No. 11, North Three-ring East Road, Chaoyang District, Beijing 100029, China

## Abstract

Psoriasis is a common autoimmune disease. Acupuncture-related techniques have been widely used to treat psoriasis since its ability to engage neuronal function, the immune system, and other systems is well documented. This study aimed to investigate and compare the effects of three common acupuncture-related techniques in psoriasis-like skin inflammatory responses and explore the possible involved mechanisms. Imiquimod (IMQ)-induced psoriasis-like mice were treated with acupuncture needling, electroacupuncture, or fire acupuncture. Methotrexate (MTX) was applied as a positive control. Scoring by the psoriasis area and severity index (PASI) evaluated skin lesion changes. Keratinocyte proliferation and inflammatory cell infiltration were investigated using pathological staining. The secretion levels of inflammatory cytokines were detected by enzyme-linked immunosorbent assay. The expression levels of neuropeptides were assessed by Western immunoblotting. We found that acupuncture needling, electroacupuncture, and fire acupuncture all ameliorated skin lesions, reduced epidermal thickness, inhibited keratinocyte proliferation, and reduced CD3^+^ T cell infiltration. The aforementioned acupuncture techniques also decreased inflammatory cytokine secretion, including IL-1*β*, IL-17A, and IL-23p40. Among them, electroacupuncture showed the best curative effects. Additionally, electroacupuncture downregulated the expression levels of Neurokinin A (NKA), which was positively associated with decreased inflammatory cytokine levels in local lesions. In conclusion, acupuncture needling, electroacupuncture, and fire acupuncture alleviated IMQ-induced psoriasis-like lesions. By contrast, electroacupuncture was more beneficial in reducing the inflammatory response, which might be related to locally dampened neuropeptide levels. Observations support the therapeutic effect of acupuncture for psoriasis and indicate a neuromodulatory mechanism in treating psoriasis by electroacupuncture.

## 1. Introduction

Psoriasis is a common chronic skin disease that is characterized by erythematous and scaly plaques. The prevalence of psoriasis is estimated at 1-3 percent of the world's population and has increased rapidly in the past 20 years in China [1]. Psoriasis is also an autoimmune disease, with immune cells like dendritic cells, T cells, macrophages, and neutrophils in the dermis and epidermis collectively playing important roles in the pathogenesis of psoriasis. These immune cells release a variety of inflammatory cytokines including IL-23, IL-1*β*, IL-6, IL-17A, and IL-22 to form a cytokine network that develops and maintains a local inflammatory response [2]. Clinical studies have demonstrated that selective inhibition of IL-23/IL-17 has utility in treating psoriasis [3].

Although many pharmacological methods are used to treat psoriasis, which include topical corticosteroids, dithranol, vitamin D analogues, oral methotrexate, cyclosporine, biologic agents, and UV phototherapy, some 52.3 percent of patients still report dissatisfaction because of treatment inefficacy and adverse effects [4]. Thus, some complementary therapies appear necessary in treating psoriasis.

Acupuncture is a complementary and alternative medicine therapy, for which clear evidence supports the efficacy of this approach in treating plaque psoriasis. A meta-analysis on acupuncture and psoriasis showed that the therapeutic effect of acupoint stimulation is superior to that of nonacupoint stimulation in treating psoriasis [5]. Acupuncture is a major Traditional Chinese Medicine technique that has been used for more than 3000 years. Since it is an effective approach, with few side-effects, this approach is now globally accepted [6].

The acupuncture procedure includes inserting needles into targeted acupoints with the intention of regulating internal organ function, and it is believed that acupuncture exerts its therapeutic effect by mediating neuronal, immune, and other systems [7, 8]. Although acupuncture appears to be beneficial in treating psoriasis, the precise immunological mechanisms remain poorly understood.

In this study, we used the classical mouse model of topically administering imiquimod (IMQ) on the back of mice to induce a psoriasis-like skin inflammation and observed the effectiveness of three types of acupuncture-related techniques (i.e., acupuncture needling, electroacupuncture, and fire needling) on the skin lesions. By these approaches, we also explored the possible underlying mechanisms.

## 2. Materials and Methods

### 2.1. Animals and Treatment

**Figure 1:**
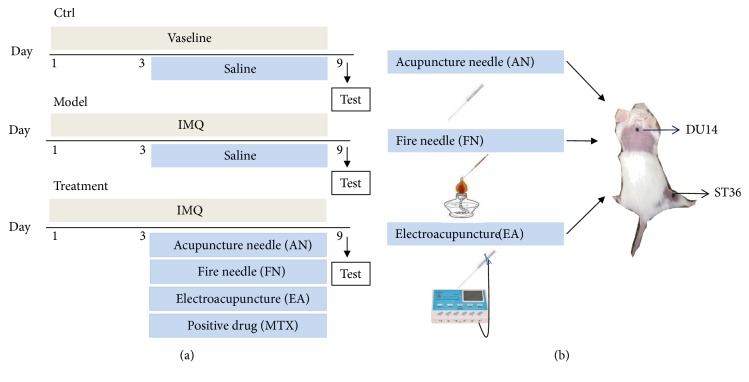
The experimental design and acupoints. (a) Detailed treatment and time schema of the experiments. (b) Acupuncture operational procedure and acupoint locations.

Male, 8-week old BALB/c mice were purchased from Beijing Huafukang Bioscience Company (experimental animal license number: SCXK Jing 2014-0013). Mice were housed under standard conditions, with free access to food and water. The back hair of experimental mice was shaved to a square-shaped area with dimensions of 2 ×2 cm^2^, following which, mice were randomly divided into the following six groups (8 mice/group) in accordance with the recommendations of the local animal ethics committee and previous experimental experience: (1)* in the control group (Ctrl)*, Vaseline (Lanlianfeitian Petrochemical Co. Ltd., China) was applied on the back for 9 days, and saline (0.4 ml/day) was administered by the intragastric route from day 4 for a total 6 days; (2)* the model group (model)* was treated with 42 mg of 5 percent IMQ cream (Mingxin Pharmaceutical co. LTD, China), which was applied on the back for 9 days, and saline (0.4 ml/day) was administered by the intragastric route from the fourth day for a total 6 days; (3)* the positive drug group (methotrexate MTX)* was treated with 42 mg of 5 percent IMQ cream that was applied on the back for 9 days, and MTX (Shanghaixinyi Pharmaceutical Co. Ltd., China) was then administered by the intragastric route at a dose of 1mg/kg/day from day 4 for a total 6 days; (4)* the acupuncture needling group (AN)* was treated with 42 mg of a 5 percent IMQ cream, which was applied on the back for 9 days, following which, acupuncture was stimulated at the Dazhui acupoint (DU14) and the right Zusanli (ST36) acupoint for 8 minutes per day from day 4, continuously for 6 days; (5)* the electroacupuncture group (EA)* was treated with 42 mg of a 5 percent IMQ cream, which was applied on the back for 9 days, following which, electroacupuncture was stimulated at the DU14 and the right ST36 acupoints for 8 minutes per day from the fourth day, continuously for 6 days (at a frequency of 2/100 Hz and a current of 10 mA to cause obvious shaking of the bodies); (6)* the fire needling group (FN)* was treated with 42 mg of a 5 percent IMQ cream, which was applied on the back for 9 days, following which, fire needling was stimulated at the DU14 and the right ST36 acupoints every other day from day 4 continuously for 6 days. The locations of the acupoints were the Dazhui acupoint (DU14, which is located in the hollow between the 7th cervical vertebra and the 1st thoracic spine) and the Zusanli acupoint (ST36, which is located below the knee joint, in the muscular groove that is located 0.3 cm below the fibula head). The detailed experimental design, operations, and acupoints are shown in Figure 1.

All animal experiments were conducted according to the National Institutes of Health Guidelines on Laboratory Research and approved by the local Animal Care and Use Committee of Capital Medical University (Beijing, China).

### 2.2. Psoriasis Area and Severity Index (PASI) Analysis

The severity of the lesions was measured every day and was based on the clinical PASI scoring system. The erythema, scaling, and infiltration were scored separately as 0 (not present); 1 (slight); 2 (moderate); 3 (severe); and 4 (extremely severe), from which, the scores were summed up for a total score.

### 2.3. Sample Preparation

The mice were sacrificed by overdosing with pentobarbital sodium, and the tissue lesions were surgically excised from the back and then divided into three tissue sections. These three tissue sections were used separately for the following observations: one part of the tissue was fixed in 10 percent formalin solution for 72h and paraffin embedded for hematoxylin and eosin (HE) staining; the remaining two parts were stored at -80°C for ELISA assays and Western immunoblotting.

### 2.4. Histological and Immunohistochemical Staining

For histological staining, the tissue sections (5 *μ*m) were cut from the paraffin sections and stained with HE for pathological observations. Histopathological changes were recorded by taking photographs by electron microscopy (Axio Imager; M2, Zeiss, Germany). Epidermal thickness was measured by an image analysis system (AxioCam HR R3 – ZEN 2 Pro, Germany). For immunohistochemical staining, the sections were stained with an anti-rabbit proliferating cell nuclear antigen (PCNA, 1:100, Abcam, Cat. No. ab15497, USA) and CD3 (1:50, Abcam, Cat. No. ab16669, USA), and the positive cells were measured by Image-Pro Plus software (Leeds Precision Instruments, Minneapolis, USA).

### 2.5. ELISA Assays

Skin tissues were weighed and homogenized using a lysis buffer. The supernatant was collected, and the expression levels of the following cytokines were measured by ELISA according to the manufacture's protocols: IL-17A (Biokits Inc., Cat. No. EM09-48/96, China), IL-1*β* (Biokits Inc., Cat. No. EM08-48/96, China), and IL-23p40 (Multi Sciences, Cat. No. EK21831/2, China).

### 2.6. Western Blotting

Total protein was extracted using a lysis buffer and the protein concentrations were quantified by the Pierce BCA Protein Assay Kit (Cat. No. 23227, Thermo Scientific, USA). Samples were separated by 10 percent sodium dodecyl sulfate-polyacrylamide gel electrophoresis (SDS-PAGE) and transferred to a nitrocellulose (NC) filter membrane. Next, the membranes were blocked in 3 percent bovine serum albumin and incubated with the following respective primary antibodies: Substance P (1:1000, Affinity, Cat. No. DF7522, USA), Neurokinin A (1:1000, Cell Signalling Technology, Cat. No. 3540, USA), and GAPDH (1:20000, ImmunoWay Biotechnology Company, Cat. No. YM3029, USA) at 4°C overnight. After washing with Tween 20 supplemented Tris-buffered saline (TBS-T), the membranes were incubated with secondary antibodies at room temperature for 40 min. Immunofluorescence was assessed using an Odyssey infrared imaging system (LI-COR Biosciences, Lincoln, NE, USA).

### 2.7. Statistical Analyses

All data were expressed as mean ± SD. One-way analysis of variance (ANOVA) was used to analyze the differences between groups. Differences were considered statistically significant at an alpha level of* p *< 0.05.

## 3. Results and Discussion

### 3.1. Acupuncture Needling, Electroacupuncture, and Fire Needling Improved Psoriasis-Like Lesions in IMQ-Induced Mice

**Figure 2:**
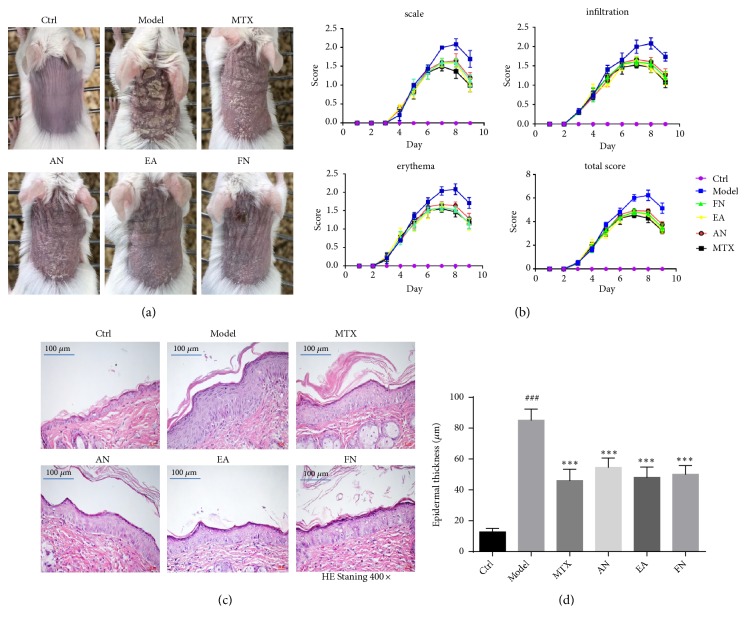
Acupuncture needling, electroacupuncture, and fire needling improved psoriasis-like lesions in IMQ mice. (a) Phenotypical presentation of the back skin on day 9 after IMQ treatment. (b) Psoriasis Area Severity Index (PASI) scoring of skin lesions, including erythema, scaling, and thickness, was scored daily on a scale from 0 to 4. The total score (erythema + scaling + thickness) was illustrated. (c) HE staining (400×). (d) The quantification of epidermal thickness of the back skin of treated mice. Scale bar = 20 *μ*m. Data are presented as the mean ± SD (n = 8 /group). ^###^*p*<0.001* vs* Ctrl; ^*∗∗∗*^*p*<0.001* vs* model.

Compared with the control mice, after topical application of IMQ for three days, psoriasis-like lesions were observed, manifested as erythema, scaling, and thickening. On day four, mice were treated with acupuncture needling, electroacupuncture, fire needling, or MTX. After treatment for two days, the psoriasis-like lesions were improved in all treatment groups of mice. After treatment for five days, the mice of all treatment groups had smoother skin, reduced erythema, and fewer scales. The morphological changes of all treatment groups are shown in Figure 2(a). The PASI scores of mice treated with acupuncture needling, electroacupuncture, fire needling, and MTX decreased as compared with the control mice (Figure 2(b)).

HE staining showed pathological changes in the skin. The model mice exhibited increased acanthosis, parakeratosis, epidermal thickening, and inflammatory infiltration. Similar to the MTX group, mice treated with acupuncture needling, electroacupuncture, and fire needling showed a smoother epidermis, decreased parakeratosis, and epidermal thickening as compared with the model group. The vertical epidermal thickness was measured using a microscope and showed that the epidermal thickness of mice in the acupuncture needling, electroacupuncture, fire needling, and MTX groups was significantly reduced (Figure 2(c)).

### 3.2. Acupuncture Needling, Electroacupuncture, and Fire Needling Alleviated Proliferation of Keratinocytes and Inflammatory Cell Infiltration in IMQ-Induced Skin Lesions

**Figure 3:**
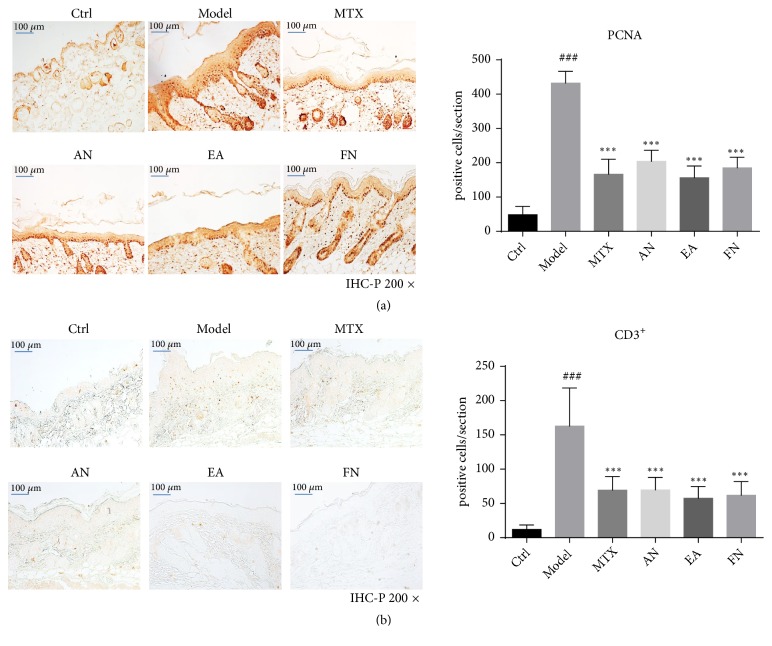
Acupuncture needling, electroacupuncture, and fire needling alleviated proliferation of keratinocytes and inflammatory cell infiltration in IMQ mice. (a) IHC staining of proliferating cell nuclear antigen (PCNA) in mouse back skin. (b) IHC staining for CD3. Data is presented as the mean ± SD (n = 6 /group). Scale bar = 50 *μ*m.

Proliferating cell nuclear antigen (PCNA) is mainly expressed in the nucleus of proliferating cells. Immunohistochemistry showed that positive cells were stained brown. The number of positive cells was significantly higher in the skin lesions of mice in the model group, while fewer were found in the skin lesions of the acupuncture needling, electroacupuncture, fire needling, and MTX groups of experimental study mice (Figure 3(a)).

The cell surface receptor CD3 is mainly expressed by T lymphocytes. We detected the effect of acupuncture needling, electroacupuncture, and fire needling on CD3^+^ T cell infiltration in the skin lesions. CD3^+^ T cells appeared as brown particles after immunohistochemistry (IHC) with a diaminobenzidine (DAB) chromogenic agent. Our results showed that the number of CD3^+^ T cells was higher in the model group and lower in the acupuncture needling, electroacupuncture, fire needling, and MTX groups (Figure 3(b)).

### 3.3. Acupuncture Needling, Electroacupuncture, and Fire Needling Reduced the Expression Levels of Inflammatory Cytokines in IMQ-Induced Skin Lesions

**Figure 4:**
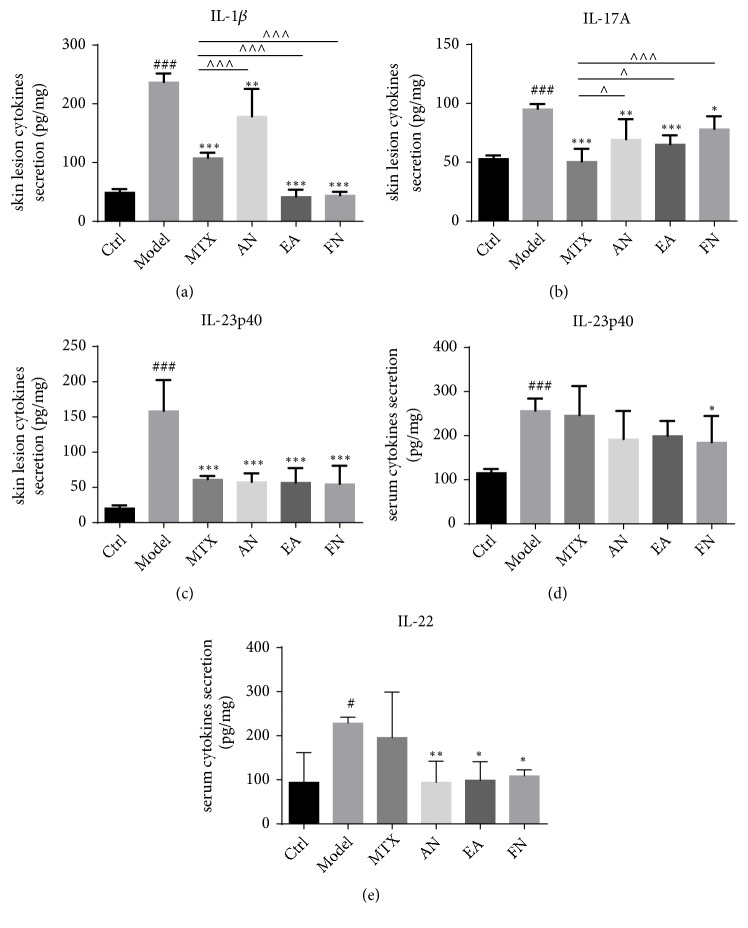
Acupuncture needling, electroacupuncture, and fire needling reduced the expression levels of inflammatory cytokines in IMQ-induced skin lesions. The expression levels of (a) IL-1*β*, (b) IL-17A, and (c) IL-23p40 in skin lesions and (d) IL-23p40 and (e) IL-22 in serum were detected by ELISA. Data are presented as the mean ± SD (n = 6 /group). ^###^*p*<0.001* vs* Ctrl; ^*∗∗*^*p*<0.01, ^*∗∗∗*^*p*<0.001* vs* model, ^∧∧∧∧^* p*<0.001, ^∧∧^* p*<0.01, ^∧^* p*<0.05* vs* MTX.

It was reported that some inflammatory cytokines like IL-17A, IL-22, IL-1*β*, and IL-12/23p40 are associated with the development of the psoriasiform dermatitis both in an IMQ-induced model and in human sufferers of the condition [9]. In this study, we determined the protein levels of these cytokines in mouse skin and serum. We found that the expression levels of IL-17A, IL-22, IL-1 *β*, and IL-23p40 in the skin lesions were increased in the model group, while the levels of IL-17A, IL-1 *β*, and IL-23p40 were significantly decreased in the acupuncture needling group, electroacupuncture group, fire needling group, and the MTX group (Figures 4(a)–4(c)). Furthermore, the serological expression levels of IL-23p40 and IL-22 were increased in the model group, while the levels of IL-23p40 were decreased in the fire acupuncture group. In addition, the levels of IL-22 were decreased in all acupuncture groups. The results also showed that mice in the electroacupuncture group displayed lower expression levels of IL-17A and IL-1*β* in the skin lesions as compared with mice in the acupuncture and fire needling groups (Figures 4(d) and 4(e)).

### 3.4. Acupuncture Needling and Electroacupuncture Reduced the Expression Levels of Neurokinin A in IMQ-Induced Skin Lesions

**Figure 5:**
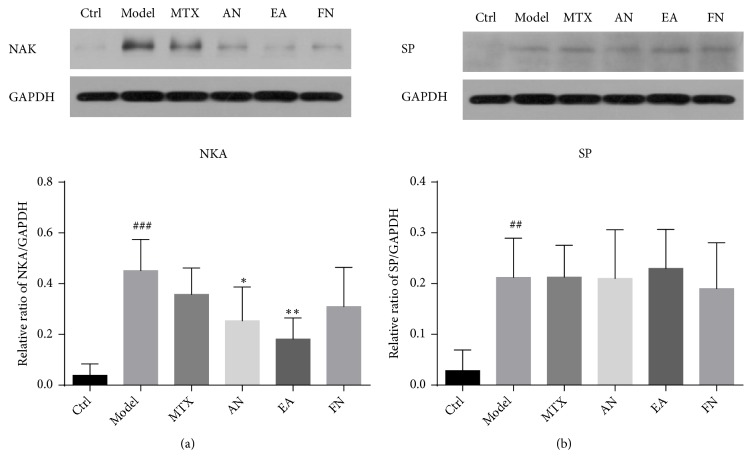
Acupuncture needling and electroacupuncture reduced the expression levels of Neurokinin A (NKA) in IMQ-induced mouse skin lesions. The expression levels of (a) NKA, and (b) Substance P (SP) were detected by Western immunoblotting. Data are presented as the mean ± SD (n = 7/group). ^##^*p*<0.01, ^###^*p*<0.001* vs* Ctrl; ^*∗*^*p*<0.05, ^*∗∗*^*p*<0.01* vs* model.

Recent studies have indicated that sensory nerve-derived peptides mediate psoriasiform dermatitis. Nerve fibers and some neuropeptides such as Substance P (SP) and Neurokinin A (NKA) are increased in the skin lesion as compared with nonlesional areas [10, 11]. Thus, we detected the expression levels of these two neuropeptides in mouse skin. We found that the expression levels of both SP and NAK were increased in the model mouse skin, while only NAK was decreased after acupuncture needling and electroacupuncture (Figures 5(a) and 5(b)).

### 3.5. Correlation Analysis between NKA Expression and Proinflammatory Cytokine Levels

**Figure 6:**
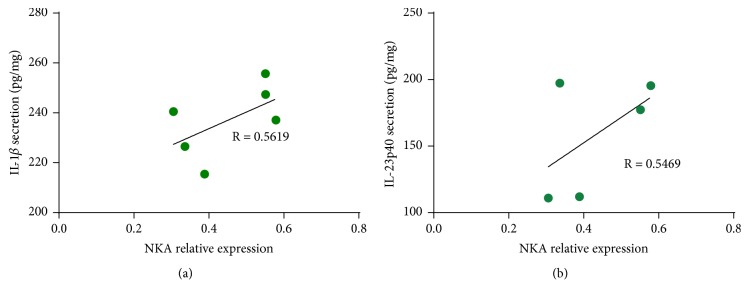
Correlation analysis of the expression levels of NKA with that of proinflammatory cytokines in the model group. (a) NKA* vs *IL-1*β*; (b) NKA* vs *IL-23p40. R: Pearson's correlation coefficient.

To explore the relationship between NKA expression and proinflammatory cytokine secretion in skin lesions, Pearson's correlation coefficient was employed. The results showed that the secretions of IL-1*β* and IL-23p40 were both positively correlated with the expression levels of NKA (Figures 6(a) and 6(b)).

## 4. Discussion

It is reported that up to 51 percent of patients with psoriasis use complementary and alternative medicine, due to dissatisfaction with their conventional medical treatment [4, 12]. Among them, acupuncture is one of the commonest methods, and acupuncture-related techniques have been used to treat psoriasis for many years, which are believed to be effective [8, 13–15]. Acupuncture, a component of Traditional Chinese Medicine, is used to stimulate so-called acupoints and modulate subsequent physiological reactions. There are several acupuncture-related techniques used in psoriasis treatment. These include needling, moxibustion, and auriculotherapy, cupping and bloodletting therapy, catgut embedding therapy, point-injection therapy, Traditional Chinese Medicine fumigation therapy, fire needling therapy, and electroacupuncture therapy [16]. Although many different acupuncture-related techniques for psoriasis have been used and widely accepted, their therapeutic effects and mechanisms of action remain unclear.

In the present study, we used an IMQ-induced psoriasis mouse model to compare the effects of needling, electroacupuncture, and fire needling and to clarify the possible mechanisms of acupuncture treatment of psoriasis.

The IMQ-induced psoriasis-like skin mouse is a classical model that closely resembles human psoriasis lesions and is used to elucidate the pathogenic mechanisms of and therapies for treating psoriasis [17]. Clinical studies showed that the most frequently used acupuncture points for psoriasis treatment are Dazhui (DU14) and Zusanli (ST36) [16, 18, 19]. Thus, we selected both of these stimulatory points using acupuncture needling, electroacupuncture, and fire needling in an IMQ-induced psoriasis-like skin mouse model and compared their therapeutic effects to also explore the underlying mechanisms.

Our findings suggested that acupuncture needling, electroacupuncture, and fire needling treatment all significantly ameliorated skin lesions and inhibited keratinocyte proliferation and proinflammatory cell infiltration. The therapeutic effects of these three methods were similar to using the positive control drug MTX; however, the MTX treatment group obtained the lowest epidermal thickness.

Psoriasis is a chronic inflammatory skin disease and can be considered an autoimmune disease. Evidence suggests that immune cells play a crucial role in the development and persistence of psoriatic lesions. T cell activation leading to keratinocyte proliferation is thought to be the central mode of action in the pathogenesis of psoriasis, as well as the therapeutic target [20]. It is believed that acupuncture therapy has a bidirectional adjustment effect on cells and tissues in the human body, and it is beneficial in the contexts of immune regulation, endocrine function, and metabolism [5].

According to existing research results, we hypothesized that the possible mechanism of acupuncture in psoriasis treatment might be due, in part, to its regulatory impact on immune cell function. It is now well recognized that the IL-23/IL-17 cytokine axis plays a central role in psoriasis, and the corresponding inhibitors showed an authentic therapeutic effect [21]. Here, we detected the expression levels of inflammatory cytokines and found that all treatment groups showed a significantly reduced level of IL-17A, IL-1*β*, and IL-23p40. Furthermore, as compared to MTX and acupuncture needling, electroacupuncture and fire needling showed a more potent ability to inhibit the expression levels of IL-1 *β*. By contrast, MTX had the strongest ability to inhibit the expression levels of IL-17 in the local skin. We also detected the expression levels of the above cytokines in serum samples and found that only IL-23p40 was decreased in the fire needling group, while IL-22 was decreased in the acupuncture, electroacupuncture, and fire needling groups; these results also supported our original hypothesis, which stated that acupuncture improved imiquimod-induced psoriasis-like skin lesions through reducing inflammatory responses.

A relatively large body of evidence from clinical and experimental studies has suggested that neurogenic components are related to the modulation of skin inflammation as well as the pathogenesis of psoriasis [22, 23]. Substance P and Neurokinin A belong to the tachykinin family and are mainly released by dermal sensory nerves. Expression of both Substance P and Neurokinin A is regulated by the secretion of particular proinflammatory cytokines including that of IL-1*β* [24]. Our research results showed that IMQ-induced psoriasis-like skin lesions had a higher level of NKA and SP. Moreover, acupuncture needling and electroacupuncture could decrease the expression levels of NKA, an effect that could be associated with improved skin inflammation. Pearson's correlation analysis showed that the expression levels of NKA were positively correlated with the levels of IL-1*β* and IL-23p40 in the model group. These findings indicated that skin inflammation was related to the expression levels of NKA in the local skin, which might represent a possible target of acupuncture therapy. In addition, we investigated the expression of vascular endothelial growth factor A (VEGFA) in the skin lesions and found that electroacupuncture showed a tendency to decrease VEGF expression. However, the differences between electroacupuncture treated animals and the model group were not significant (data not shown). Clearly, additional experiments are needed to explain the exact mechanisms that are involved.

## 5. Conclusions

Our study suggests that acupuncture needling, electroacupuncture, and fire needling ameliorate the skin lesions in an IMQ-induced psoriasis-like murine model. We found that electroacupuncture was comparatively more beneficial in reducing inflammatory responses, which might be related to decreases in the local levels of neuropeptide. These findings provide novel insights into the therapeutic effect and neuromodulatory mechanism of acupuncture in the setting of psoriasis and other inflammatory-associated skin diseases.

## Data Availability

The data used to support the findings of this study are available from the corresponding author upon request.
